# Telemedicine in Pediatric Cardiology: Current Applications, Clinical Impact, and Future Perspectives

**DOI:** 10.3390/jpm16090453

**Published:** 2026-08-28

**Authors:** Luisa M. Rizzo, Matilde Petz, Federico Carlini, Susanna Esposito

**Affiliations:** Pediatric Clinic, Department of Medicine and Surgery, University of Parma, 43126 Parma, Italy; luisa.rizzo@unipr.it (L.M.R.); matilde.petz@unipr.it (M.P.); federico.carlini@unipr.it (F.C.)

**Keywords:** telemedicine, pediatric cardiology, congenital heart disease, tele-echocardiography, remote monitoring, wearable devices

## Abstract

Telemedicine is increasingly transforming pediatric cardiology by expanding access to specialized care, supporting early diagnosis, and improving longitudinal monitoring of children with cardiovascular disease. This narrative review summarizes current applications of digital health in pediatric cardiology, with emphasis on congenital heart disease, pediatric hypertension, and arrhythmia management. Tele-echocardiography represents one of the most established telehealth tools, enabling remote interpretation of fetal, neonatal, and pediatric echocardiographic images and improving referral appropriateness, particularly in peripheral or resource-limited settings. In infants with complex congenital heart disease, especially those with single-ventricle physiology during the interstage period, home monitoring programs using mobile applications, pulse oximeters, digital scales, and structured caregiver reporting may facilitate early recognition of clinical deterioration and reduce avoidable transfers. In non-congenital cardiovascular disease, home blood pressure monitoring can improve diagnostic accuracy by reducing white-coat effects and supporting repeated measurements in real-life settings. Smartphone-enabled electrocardiographic devices and wearable technologies may enhance detection of intermittent arrhythmias and strengthen outpatient management. Despite these advantages, challenges remain, including data fragmentation, limited pediatric validation of consumer devices, interoperability issues, privacy concerns, and socioeconomic disparities in technology access. Properly integrated telemedicine may promote more timely, equitable, and patient-centered pediatric cardiovascular care.

## 1. Introduction

Pediatric cardiology is a highly specialized field in which expertise is frequently concentrated in tertiary referral centers, while affected children and their families may live considerable distances from specialist services. Telemedicine offers a means of extending specialist expertise beyond the hospital by supporting remote diagnosis, monitoring, consultation, and longitudinal follow-up. Its potential applications are particularly relevant to congenital heart disease (CHD), in which timely diagnosis and surveillance can influence clinical management, but increasingly extend to acquired cardiovascular conditions, hypertension, and rhythm disorders.

For clarity, in this review, telemedicine refers to the delivery of clinical care at a distance using communication technologies; telehealth is used as the broader term encompassing telemedicine together with remote education, monitoring, and other digitally supported healthcare activities; and digital health refers more broadly to technologies such as mobile applications, wearable sensors, connected medical devices, and digital data platforms. These concepts overlap in contemporary practice, and the term telemedicine is used when referring specifically to remote clinical interactions.

Current pediatric applications include fetal and neonatal tele-echocardiography, home monitoring of infants with complex CHD, virtual follow-up, remote blood pressure assessment, smartphone-enabled electrocardiography, and wearable rhythm monitoring. International scientific societies have recognized the clinical utility of selected applications, particularly tele-echocardiography, when appropriate technical and clinical standards are followed [1,2]. However, the evidence base is heterogeneous: some approaches are supported by guidelines and substantial clinical experience, whereas others remain based mainly on observational studies, small cohorts, feasibility studies, or technologies developed primarily for adults.

Table 1 summarizes the main clinical applications of telemedicine in pediatric cardiology.

This narrative review summarizes current applications of telemedicine in pediatric cardiology, focusing on areas with an identifiable pediatric evidence base, including tele-echocardiography, remote monitoring of children with CHD, home blood pressure monitoring, and digital tools for arrhythmia detection. Particular attention is given to the strength and limitations of the available evidence, practical implementation, equity, cost considerations, and future integration of artificial intelligence and interoperable digital-health systems.

## 2. Methods

A narrative literature review was performed to identify relevant evidence regarding telemedicine and digital-health applications in pediatric cardiology. Searches were conducted in PubMed, Scopus, and the Cochrane Library for articles published between January 2010 and December 2025. Earlier landmark studies were additionally considered when they provided important historical or methodological information that remained relevant to current practice.

Search terms included combinations of keywords and Medical Subject Headings (MeSH), where applicable, including “telemedicine,” “telehealth,” “digital health,” “remote monitoring,” “pediatric cardiology,” “congenital heart disease,” “acquired heart disease,” “tele-echocardiography,” “home monitoring,” “blood pressure monitoring,” “wearable devices,” “electrocardiography,” “arrhythmia detection,” and related terms. Boolean operators AND and OR were used to combine concepts and adapt the searches to the individual databases.

Studies were considered eligible when they: (1) included children or adolescents younger than 18 years, or reported pediatric data separately from mixed populations; (2) addressed congenital or acquired cardiovascular disease; and (3) evaluated or discussed a telemedicine- or digital-health intervention relevant to pediatric cardiology, including teleconsultation, tele-echocardiography, home monitoring, connected physiological devices, mobile-health applications, or wearable technologies. Observational studies, prospective and retrospective cohorts, randomized and non-randomized clinical studies, systematic and narrative reviews, consensus documents, scientific statements, and relevant clinical guidelines were considered. Studies focusing exclusively on adults, non-cardiovascular conditions, or digital interventions without relevance to pediatric cardiovascular care were excluded.

Titles and abstracts identified through the searches were screened for relevance, followed by full-text assessment of potentially eligible publications. Reference lists of relevant reviews, guidelines, and selected studies were also examined to identify additional publications. Because this was a narrative rather than a systematic review, study selection was guided by relevance to the predefined clinical themes rather than by a formal systematic-review protocol.

No formal risk-of-bias tool or evidence-grading framework was applied. Nevertheless, interpretation of the evidence considered study design, sample size, pediatric specificity, single- versus multicenter setting, duration of follow-up, and type of outcome evaluated. Findings from professional guidelines, systematic reviews, prospective studies, and multicenter cohorts were distinguished from those derived predominantly from retrospective, single-center, feasibility, or proof-of-concept studies. Particular caution was applied to evidence extrapolated from adult populations and to commercially available technologies without specific pediatric validation.

The literature was synthesized qualitatively according to the major clinical applications of telemedicine in pediatric cardiology. Given the narrative design, this review has inherent limitations, including the possibility of selection bias, absence of formal quantitative synthesis, and lack of systematic risk-of-bias assessment. The conclusions should therefore be interpreted as a clinically oriented synthesis of the available literature rather than as formal evidence-graded recommendations.

## 3. Telehealth Solutions for Patients with Congenital Heart Disease

Congenital heart disease (CHD) represents one of the main areas in which telemedicine has been integrated into pediatric cardiology practice. Digital health tools are increasingly used across different stages of care, from fetal and neonatal diagnosis to postoperative surveillance and long-term follow-up. In children with CHD, telehealth solutions may be particularly valuable because clinical expertise is often concentrated in tertiary referral centers, whereas patients and families may live far from specialized services. In this setting, telemedicine can support early diagnosis, improve referral appropriateness, enhance continuity of care, and reduce unnecessary transfers or hospital visits.

The main applications of telemedicine in CHD include neonatal and fetal triage through tele-echocardiography, high-risk infant home monitoring programs during the surgical interstage period, and remote follow-up of children and adolescents with complex cardiac conditions [3,4,5]. Available evidence suggests that these approaches may improve clinical outcomes and optimize healthcare resource use, particularly when embedded within structured hub-and-spoke networks. However, broader implementation still requires the resolution of several barriers, including fragmentation of data collection and retrieval, limited validation of pediatric-specific digital tools, heterogeneous protocols, and socioeconomic disparities in access to technology [6].

Advances in surgical, interventional, and intensive care management have markedly improved survival in patients with CHD, with most affected infants now reaching adolescence and adulthood. As survival improves, the burden of long-term cardiovascular morbidity also increases, requiring lifelong surveillance for residual lesions, ventricular dysfunction, hemodynamic deterioration, and arrhythmias. Telemedicine may help tertiary pediatric cardiology centers extend their clinical reach into local communities and patients’ homes, supporting early triage, proactive intervention, and longitudinal multidisciplinary care [7].

The strength of evidence differs substantially among individual telemedicine applications. Tele-echocardiography is supported by professional guidance and substantial observational experience, whereas evidence for many newer home-monitoring and wearable technologies remains dominated by retrospective cohorts, single-center studies, feasibility investigations, and heterogeneous digital platforms. Accordingly, outcomes such as technical feasibility, diagnostic concordance, improved communication, or reduced travel should not be interpreted as equivalent to evidence of reductions in morbidity or mortality. In complex home-monitoring programs, the independent contribution of the digital technology is also difficult to isolate because interventions frequently combine caregiver education, structured surveillance, predefined escalation criteria, and enhanced access to specialist teams.

### 3.1. Tele-Echocardiography

Tele-echocardiography is one of the most established applications of telemedicine in pediatric cardiology. It allows echocardiographic images to be acquired in peripheral or spoke centers and interpreted remotely by expert pediatric cardiologists located in tertiary hub centers. This model is particularly relevant in neonatal and pediatric populations, in which early recognition of CHD is essential, but access to on-site pediatric cardiology expertise may be limited [8,9].

Tele-echocardiography can also be applied during fetal life, improving access to prenatal cardiac assessment and facilitating the detection of critical CHD, especially in peripheral, rural, or resource-limited settings. Early prenatal diagnosis enables appropriate planning of delivery in specialized centers, timely postnatal management, and, when needed, the treatment and follow-up of fetal arrhythmias. In this context, telemedicine may contribute to more coordinated perinatal care and potentially improve clinical outcomes [10].

In neonatal and pediatric practice, tele-echocardiography is mainly used for screening, initial evaluation of suspected CHD, and clinical decision support in Level I–II Neonatal Intensive Care Units (NICUs) without on-site pediatric cardiology services. Two main operational models are available: asynchronous, or store-and-forward, systems, in which recorded images are transmitted for later interpretation; and synchronous systems, which allow real-time interaction between the local operator and the remote pediatric cardiologist during image acquisition. Several studies have shown that tele-echocardiography is feasible and diagnostically reliable for identifying clinically significant CHD [1]. Its implementation may improve the appropriateness of referrals to tertiary centers, reduce unnecessary neonatal transfers, and generate organizational and economic benefits.

Makkar et al. evaluated a hybrid tele-echocardiography system for CHD screening in a Level II NICU without on-site pediatric cardiology expertise. The system improved the timeliness of clinical decision-making, enabling early identification of neonates requiring transfer to tertiary care while reducing unnecessary transfers. These findings support the use of hybrid telemedicine models to optimize resource allocation and improve access to specialized expertise in peripheral or resource-limited settings [8]. Similarly, McCrossan et al. reported an eight-year experience in which tele-echocardiography enabled a definitive diagnosis in 97% of neonates, while transfer to a higher-level center was avoided in 95 patients, corresponding to 72% of cases [11].

An important development in neonatal care has been the increasing availability of neonatologists with specific training and competency in functional neonatal echocardiography, also referred to in some settings as targeted neonatal echocardiography. Structured training programs, courses, and dedicated educational resources have enabled appropriately trained neonatologists to use echocardiography for assessment of neonatal cardiovascular physiology at the bedside [12]. Importantly, such training also includes recognition of findings outside the scope of functional assessment and identification of infants in whom congenital structural heart disease is suspected and formal pediatric cardiology assessment is required. Clinical scenarios such as persistent or oxygen-resistant cyanosis, an abnormal cardiac anatomy or function, or other findings suggestive of critical CHD should prompt timely pediatric cardiology involvement [12].

These trained neonatal clinicians may therefore play a particularly valuable role within tele-echocardiography networks. They can acquire focused and technically appropriate images at the referring center, discuss physiological and anatomical findings with the remote pediatric cardiologist in real time, and obtain additional views under specialist guidance when necessary. This collaboration can strengthen the hub-and-spoke model by combining local neonatal expertise with remote specialist pediatric cardiology interpretation while maintaining clear referral pathways for abnormalities requiring comprehensive echocardiographic evaluation.

Despite these advantages, the effectiveness of tele-echocardiography depends on several factors, including the quality of image acquisition, the training and experience of local personnel, the availability of adequate technological infrastructure, and the use of standardized acquisition and reporting protocols. Current limitations include variability among protocols, limited interoperability between systems, and the relative scarcity of prospective multicenter studies assessing long-term clinical outcomes [3,10,13].

The principal tele-echocardiography models, together with their advantages and limitations, are summarized in Table 2.

### 3.2. High-Risk Infant Home Monitoring Programs

High-risk infant home monitoring (IHM) programs represent another major application of digital health in pediatric cardiology. These programs are most commonly used during the surgical interstage period in infants with single-ventricle physiology, such as hypoplastic left heart syndrome or tricuspid atresia. The interstage period, between the first palliative procedure, such as the Norwood operation or a hybrid approach, and the second-stage Glenn procedure or superior cavopulmonary anastomosis, is one of the most vulnerable phases in the care of children with complex CHD [14,15].

During this period, infants remain at high risk of clinical deterioration because systemic and pulmonary blood flow depend on a delicate hemodynamic balance. This balance may be maintained by a surgical shunt, such as a Blalock-Taussig-Thomas shunt or a Sano shunt, or by a patent ductus arteriosus, structures that may be vulnerable to obstruction, thrombosis, or changes in vascular resistance [13,16]. For this reason, timely recognition of early warning signs is crucial.

Before hospital discharge, caregivers are usually trained to perform daily monitoring at home using structured protocols and, increasingly, digital tools. These may include dedicated mobile applications, digital scales, and Bluetooth-enabled pulse oximeters. The parameters most commonly monitored include oxygen saturation, body weight, and nutritional intake. Oxygen saturation provides information on shunt patency and the balance between pulmonary and systemic blood flow; body weight helps detect poor growth, dehydration, or fluid retention; and caloric and fluid intake allow assessment of feeding tolerance and energy balance [14,17,18].

Patient-generated health data can be transmitted in real time, or at predefined intervals, to tertiary pediatric cardiology teams, allowing early triage and proactive clinical intervention when concerning trends emerge. Evidence suggests that structured IHM programs may reduce interstage mortality, support earlier recognition of clinical deterioration, decrease inappropriate emergency transfers, and improve healthcare resource utilization [19]. However, universal implementation remains challenging because of data fragmentation, differences in monitoring protocols, limited interoperability between platforms, and socioeconomic or geographic disparities in access to digital technologies.

With technological progress, traditional paper-based or telephone-based monitoring strategies have increasingly evolved toward application-based remote monitoring systems. These platforms allow caregivers to enter key physiological parameters through smartphones or tablets, often with automated alerts and longitudinal visualization of clinical trends. Observational studies indicate that application-based systems are feasible, well accepted by families, and associated with high adherence to daily data entry and improved communication between caregivers and healthcare providers [20]. Such tools may also standardize care pathways and reduce caregiver burden compared with less integrated monitoring approaches [21].

Nevertheless, most available studies remain limited by single-center design, retrospective methodology, small sample sizes, and heterogeneity in the technologies and protocols used. Therefore, application-based IHM should currently be considered a valuable adjunct to structured clinical programs rather than a replacement for in-person evaluation and specialist follow-up. Future multicenter prospective studies are needed to define standardized protocols, validate pediatric-specific digital tools, assess cost-effectiveness, and ensure equitable access for all families affected by complex CHD.

## 4. Telehealth Solutions for Patients with Non-Congenital Heart Disease

Although telemedicine has been widely applied in congenital heart disease, its role is increasingly expanding to children and adolescents with non-congenital cardiovascular conditions. In this setting, digital health tools may support diagnosis, follow-up, and long-term risk stratification, particularly for conditions characterized by intermittent findings, variable clinical expression, or the need for repeated measurements over time. Among these, pediatric hypertension and arrhythmias represent two major areas in which telehealth-based approaches have shown growing clinical relevance.

### 4.1. Home Blood Pressure Monitoring in Children and Adolescents

In recent years, increasing attention has been directed toward pediatric arterial hypertension, whose prevalence is rising worldwide, largely in parallel with the growing burden of childhood overweight and obesity. Accurate diagnosis remains challenging because blood pressure values in children are influenced by age, sex, height, emotional state, measurement technique, and environmental factors. In this context, telemedicine and digital health may help overcome one of the main limitations of pediatric blood pressure assessment: the unreliability of isolated office or hospital measurements [22].

Home blood pressure monitoring allows repeated measurements to be obtained in a familiar environment, reducing the influence of anxiety and improving the assessment of true blood pressure patterns over time. Current recommendations emphasize the importance of correct measurement technique, validated devices, appropriate cuff size, and repeated readings when evaluating blood pressure in children and adolescents [23,24,25]. Compared with single in-clinic measurements, home monitoring may improve diagnostic accuracy, support the identification of white-coat hypertension or masked hypertension, and facilitate safer follow-up after lifestyle or pharmacological interventions.

The usefulness of remote home blood pressure monitoring is supported by recent evidence. Studies evaluating telemedicine-based home monitoring have shown that repeated digital measurements may reduce diagnostic error and improve adherence to follow-up. In particular, home-based strategies may help identify or exclude the white-coat effect, which can lead to overestimation of cardiovascular risk and unnecessary treatment when blood pressure is assessed only in clinical settings [23]. Moreover, caregiver-performed measurements using automatic oscillometric devices and dedicated applications have shown good feasibility and concordance with measurements obtained by trained healthcare professionals, supporting the reliability of remote blood pressure monitoring in children and adolescents aged 3–17 years [24].

Digital platforms may further improve pediatric blood pressure assessment by integrating automated algorithms that calculate percentiles according to age, sex, and height. This is particularly relevant because interpretation of pediatric blood pressure values is more complex than in adults and requires comparison with normative percentile tables. Applications capable of rapidly classifying blood pressure values may support both primary care pediatricians and families, facilitating early identification of children who require further evaluation or specialist referral [25,26].

The clinical importance of early recognition of pediatric hypertension is underscored by evidence linking elevated blood pressure in childhood with increased risk of cardiovascular and renal complications later in life. Children with undiagnosed or untreated hypertension may be at higher risk of adverse cardiovascular outcomes in young adulthood, including myocardial infarction, stroke, and chronic kidney disease. Therefore, telemedicine-supported home blood pressure monitoring should be regarded as a valuable tool for early detection, longitudinal surveillance, and prevention, particularly when embedded within structured clinical pathways and combined with appropriate medical interpretation.

### 4.2. Arrhythmias

Cardiac arrhythmias are an important cause of morbidity in children and adolescents. Their diagnosis may be particularly challenging because many rhythm disturbances are paroxysmal and may not occur during a conventional clinical evaluation or short period of ambulatory monitoring. This issue is especially relevant in the investigation of recurrent unexplained syncope, presyncope, or infrequent palpitations, in which establishing a symptom–rhythm correlation is central to determining whether an arrhythmic mechanism is present. Traditional diagnostic strategies include 24 h or extended Holter monitoring and external event recorders. When clinically significant symptoms are infrequent and conventional monitoring is unrevealing, implantable loop recorders (ILRs) can provide much longer-term continuous rhythm surveillance and may substantially increase the opportunity to document the cardiac rhythm at the time of a spontaneous event [27]. Contemporary miniaturized ILRs, such as the Reveal LINQ system and similar devices, can also support remote transmission of recorded episodes, integrating long-term diagnostic monitoring with telemedicine-based follow-up.

Telemedicine and remote monitoring have introduced new opportunities for arrhythmia detection and management. In children and young adults with congenital or acquired heart disease who carry pacemakers or implantable cardioverter defibrillators, home monitoring systems can automatically transmit device data to the clinical team. This allows earlier identification of device malfunction, arrhythmic events, or clinically relevant changes, without requiring active intervention by the patient or caregiver [28,29]. Such systems may improve surveillance, reduce unnecessary hospital visits, and support timely therapeutic adjustments.

Smartphone-enabled ECG devices have also shown promising results in pediatric outpatient arrhythmia management. Gropler et al. evaluated the comparability of electrocardiographic intervals recorded with the AliveCor Kardia Mobile device and those obtained using a standard 12-lead ECG in pediatric patients. Their findings suggested that heart rate, rhythm, and ECG intervals can be accurately assessed using this wireless device in children with and without congenital heart disease or arrhythmias [30]. Similarly, Nguyen et al. demonstrated that smartphone-enabled ECG recordings can provide diagnostic-quality tracings in children with atrial fibrillation and supraventricular tachycardia, including at heart rates exceeding 200 beats per minute, thereby supporting remote clinical assessment and management [28].

These tools may also be useful in community and low-resource settings. Shepherd et al. described the successful use of a Kardia monitor to assess a pediatric patient with suspected arrhythmia in a remote coronavirus assessment center. In that case, the device was delivered to the patient’s mother, who was able to record an ECG tracing and transmit it to both the assessment center and the pediatric specialist, minimizing the need for direct physical contact and facilitating timely evaluation [31,32]. Overall, smartphone-based ECG devices may help reduce emergency department visits, improve symptom-rhythm correlation, and support earlier diagnosis of intermittent arrhythmias.

The rapid spread of wearable devices has further changed the diagnostic paradigm in arrhythmia care. Increasingly, patients and families are able to detect rhythm abnormalities and share recordings with healthcare professionals, rather than relying exclusively on physician-initiated monitoring [33]. Smartwatches are among the most widely used devices in this field. Most use photoplethysmography to detect pulse irregularities and may prompt users to record a single-lead ECG when an arrhythmia is suspected. Devices such as the Apple Watch and Samsung Galaxy Watch can generate ECG tracings that may provide clinically useful information and support more timely decision-making [34,35,36,37].

In pediatric populations, Kobel et al. evaluated the use of Apple Watch iECG and reported good comparability between lead I of the standard 12-lead ECG and the smartwatch-derived tracing, both in terms of amplitude and interval measurements. The recordings were also considered suitable for rhythm classification, including in children with congenital heart disease, a population at increased risk of arrhythmias [35]. These findings suggest that smartwatch-based ECG may represent a useful adjunct in selected pediatric patients, particularly for documenting intermittent symptoms.

An important distinction should therefore be made between wearable consumer technologies and continuous or near-continuous diagnostic monitoring. Smartwatches and smartphone-enabled ECG systems are valuable for obtaining an ECG during symptoms when the patient or caregiver is able to activate the device; however, they may fail to document the onset of a sudden arrhythmia and may be of limited value when syncope results in immediate loss of consciousness or prevents device activation. In selected patients with recurrent unexplained syncope or very infrequent suspected paroxysmal arrhythmias, prolonged continuous monitoring with an implantable loop recorders (ILR) may therefore provide information that patient-triggered wearable recordings cannot capture [27]. Consumer wearables and ILRs should consequently be considered complementary rather than interchangeable technologies, with device selection determined by symptom frequency, clinical risk, and the diagnostic question.

Other wearable technologies are also being investigated. Smart rings, for example, use photoplethysmography to measure physiological parameters from a highly vascularized anatomical site; however, most commercially available smart rings do not currently provide ECG tracings. Patch monitors and textile-based systems, such as smart shirts equipped with ECG sensors, may represent alternatives to conventional Holter monitoring. Patch ECG devices can provide prolonged monitoring and have shown higher arrhythmia detection rates than standard 24 h Holter recordings in selected settings. Smart shirts may offer signal quality comparable to traditional Holter devices, with the additional advantage of improved comfort, which may increase adherence in children and adolescents [37].

Despite their potential, consumer and wearable technologies also raise important concerns. Many devices become widely available before undergoing rigorous validation in pediatric populations, and even approved devices are often developed for adults or for general wellness rather than disease-specific management. Therefore, their use in children requires careful clinical interpretation, attention to false positives and false negatives, and integration into defined care pathways. Further challenges include data overload, interoperability with hospital systems, privacy and security issues, unequal access to technology, and the need for clinician training [34,35].

An important distinction should be made between clinically validated monitoring devices and consumer-grade wearable technologies. Medical ECG recorders, implantable rhythm monitors, and some ambulatory patch systems are designed for diagnostic use and are subject to regulatory and clinical validation requirements. By contrast, many smartwatches, smart rings, and wellness-oriented wearables are primarily consumer devices, although selected models incorporate regulated ECG functions. Validation of an individual function should not be interpreted as validation of the entire device for diagnosis or management of pediatric arrhythmias.

This distinction is particularly important in children because many algorithms were developed and tested predominantly in adult populations. Age-related differences in heart rate, small body size, movement artifact, device fit, and the ability of young children to activate recordings may affect performance. Consumer-generated recordings may provide valuable supplementary information, particularly when symptoms are intermittent, but false-positive findings can cause anxiety and unnecessary investigations, whereas false-negative or technically inadequate recordings may provide inappropriate reassurance. Consumer wearable technologies should therefore complement, rather than replace, conventional diagnostic testing and pediatric electrophysiological assessment when these are clinically indicated.

That develops a caution your existing manuscript already contains: it notes that many devices reach the market without rigorous pediatric validation and identifies false positives, false negatives, privacy, data overload, and interoperability as concerns.

Overall, telemedicine-based arrhythmia tools should be considered complementary to conventional diagnostic strategies rather than replacements for specialist evaluation. When appropriately validated and integrated into clinical workflows, smartphone ECG devices, remote monitoring platforms, and wearables may improve arrhythmia detection, strengthen outpatient management, reduce unnecessary emergency visits, and promote more personalized care for children and adolescents with rhythm disorders.

Table 3 summarizes the main digital tools currently used or under investigation for home monitoring and arrhythmia detection in pediatric populations.

## 5. Critical Appraisal of the Evidence, Implementation Challenges, and Research Priorities

The available evidence supporting telemedicine in pediatric cardiology is heterogeneous in both methodological quality and clinical maturity. Although telemedicine is increasingly incorporated into clinical practice, evidence of technical feasibility should be distinguished from evidence of improved clinical outcomes. Tele-echocardiography represents one of the more established applications, supported by professional recommendations, long-term clinical experience, and observational studies demonstrating reliable remote interpretation and improved referral pathways. Nevertheless, much of the literature remains derived from single-center or regional experiences, while prospective multicenter studies assessing long-term patient-centered outcomes are relatively limited. Accordingly, successful image transmission, diagnostic concordance, reduced travel, or avoidance of transfer should not automatically be interpreted as evidence of reductions in morbidity, mortality, or overall healthcare expenditure. Technical feasibility, diagnostic performance, healthcare utilization, and clinical effectiveness represent distinct outcomes and should be interpreted separately.

A similar consideration applies to remote home-monitoring programs for children with CHD. Interstage surveillance of infants with single-ventricle physiology is among the most clinically developed pediatric applications, and several studies have associated structured monitoring programs with earlier recognition of deterioration and improved outcomes. However, these programs are complex interventions rather than isolated technological solutions. They often combine caregiver education, pulse oximetry, body-weight and nutritional monitoring, mobile or telephone communication, predefined warning thresholds, and rapid access to specialized cardiac teams. Consequently, favorable outcomes cannot necessarily be attributed to the digital component alone. Confounding by concurrent improvements in clinical organization, surveillance intensity, caregiver education, and specialist availability remains difficult to exclude, particularly in observational studies. Selection bias is also possible because families who successfully participate in intensive home-monitoring programs may differ from non-participants in socioeconomic resources, digital literacy, caregiver availability, access to technology, and ability to adhere to monitoring protocols.

Recent pediatric evidence confirms both the expanding use of remote-monitoring technologies and the persistence of these methodological limitations. In a 2024 systematic review, Duffy et al. identified a growing range of wearable and remote-monitoring technologies for children with CHD but emphasized that pediatric development and validation continue to lag behind adult applications [38]. Similarly, Schöneburg et al. reviewed interactive telehealth approaches in CHD and identified substantial heterogeneity in interventions, study populations, designs, and reported outcomes, limiting direct comparison between studies and highlighting the need for more standardized evaluation [39]. More broadly, Exner et al. reviewed prospective telemedicine and digital-health interventions across multiple pediatric chronic diseases and showed that research remains concentrated in a limited number of conditions, while many pediatric specialties, including cardiology, are less extensively represented [40].

A major challenge is the extent to which evidence generated in adults can be extrapolated to children. Many commercially available wearable devices, rhythm-detection algorithms, mobile applications, and remote-monitoring technologies were initially developed and validated in adult populations. Pediatric application may be affected by age-dependent heart rates, smaller body size, motion artifact, differences in arrhythmia prevalence, device fit, and the ability of younger children to recognize symptoms or activate recordings. Consumer devices may generate false-positive alerts, potentially leading to anxiety, unnecessary specialist referrals, additional diagnostic testing, and increased healthcare utilization. Conversely, false-negative or technically inadequate recordings may provide inappropriate reassurance and delay conventional assessment. Consumer-generated cardiovascular data should therefore be regarded as complementary to, rather than a substitute for, clinically indicated diagnostic testing and specialist pediatric cardiology evaluation.

The concentration of published evidence in CHD, tele-echocardiography, arrhythmia monitoring, and pediatric hypertension should also be interpreted cautiously. This distribution does not imply that telemedicine has limited value in other pediatric cardiovascular conditions. Rather, these areas are particularly suited to remote evaluation because they involve physiological, electrical, or imaging parameters that can be measured and transmitted electronically. Potential future applications include pediatric heart failure, cardiomyopathy, pulmonary hypertension, cardiac transplantation, cardio-oncology, rehabilitation, exercise prescription, and transition from pediatric to adult congenital heart disease care. However, pediatric-specific evidence in these areas remains substantially less developed. Several factors may contribute to this gap, including the relative rarity and heterogeneity of many pediatric cardiac disorders, the difficulty of recruiting adequately powered cohorts at single centers, differences in digital infrastructure and clinical pathways across institutions, dependence on caregivers for technology use, and the rapid evolution of digital devices, which may outpace the time required to conduct long-term prospective trials.

### 5.1. Real-World Implementation, Cost-Effectiveness, and Healthcare Policy

Real-world effectiveness depends on factors that may not be fully captured by studies performed in highly organized tertiary centers. Telemedicine requires investment in hardware, software, secure communication platforms, cybersecurity, technical support, data integration, staff training, maintenance, and clinical time devoted to reviewing remotely generated information. Therefore, reductions in patient travel or hospital transfer should not automatically be interpreted as overall cost savings.

Early economic evaluations of pediatric telecardiology illustrate this point. Sicotte et al. reported that interactive telecardiology reduced patient journeys but was associated with higher overall health-system costs because of equipment and telecommunications expenditure [41]. Dowie et al. similarly demonstrated that the economic value of pediatric and perinatal telecardiology depended substantially on organizational context and patterns of service utilization [42]. These studies were conducted using technologies that differ markedly from contemporary digital platforms, and their absolute cost estimates are unlikely to be directly transferable to current practice. Nevertheless, they remain informative because they demonstrate that economic value depends on the entire care pathway rather than simply on whether a consultation is conducted remotely.

More recent pediatric experience suggests that economic effects vary according to the clinical model. Adroher Mas et al. reported lower daily costs for pediatric tele-home care compared with conventional hospital care in a cost-minimization analysis [43]. This suggests that digitally supported care may provide economic benefit in selected settings, but such findings should not be generalized to all forms of telemedicine. Future evaluations should include implementation and maintenance costs, software licensing, equipment replacement, clinician and technical-support workload, downstream investigations generated by remote alerts, emergency department use, hospital admission, and interhospital transfers. From the family and societal perspectives, relevant outcomes include transportation costs, time away from employment or school, childcare requirements, accommodation expenses, and caregiver burden.

Healthcare policy will have an equally important role in determining whether telemedicine can progress from individual pilot programs to sustainable routine care. Clear governance frameworks are required for reimbursement, professional responsibility, licensing and credentialing, informed consent, data protection, cybersecurity, documentation standards, medical-device regulation, and cross-institutional data sharing. Policies should also define who is responsible for reviewing remotely generated information, expected response times for abnormal alerts, and criteria for escalation to urgent or face-to-face assessment. Without clear reimbursement and governance structures, otherwise effective programs may remain dependent on short-term research funding or local institutional initiatives.

### 5.2. Equity and Digital Inclusion

Equity is another major consideration. Telemedicine has the potential to improve access for children who live far from specialist pediatric cardiology centers, but digital delivery may also create or amplify disparities. Families with unreliable broadband, limited access to smartphones or computers, lower digital literacy, language barriers, unstable housing, disability-related technology needs, or limited caregiver availability may be less able to participate in remote care.

Gilkes et al. evaluated a pediatric program providing tablets and Wi-Fi hotspots to socioeconomically disadvantaged families and found that most surveyed caregivers reported that the devices enabled telehealth participation; however, substantial device disuse occurred during longer-term follow-up [44]. These findings suggest that provision of equipment alone does not guarantee sustainable digital inclusion. Recent pediatric specialty-care data have also demonstrated differences in telemedicine uptake according to demographic and language-related factors [45].

Selection bias should therefore be considered when interpreting reports of feasibility, acceptability, or satisfaction. Families who agree to participate in telemedicine research may have greater digital confidence, higher educational attainment, or stronger engagement with healthcare services than the broader population. Future studies should report not only outcomes among successful users but also the proportion and characteristics of families who decline participation, cannot establish a digital connection, discontinue monitoring, or require conversion to conventional care. Equity should be treated as a measurable implementation outcome rather than assumed to be an intrinsic benefit of telemedicine.

### 5.3. Artificial Intelligence, Predictive Analytics, and Integration with Electronic Health Records

An important future direction for pediatric telecardiology is the integration of artificial intelligence (AI), predictive analytics, and interoperable electronic health records (EHRs). Remote-monitoring programs increasingly generate longitudinal datasets that may include heart rate and rhythm, oxygen saturation, blood pressure, body weight, activity, symptoms, feeding parameters, echocardiographic images, and caregiver-reported information. At present, much of this information is interpreted manually or through predefined threshold-based alerts. AI-based approaches may allow multiple data streams to be analyzed simultaneously, potentially identifying subtle patterns of deterioration before conventional warning thresholds are reached.

Potential applications include automated or semi-automated ECG interpretation, arrhythmia classification, echocardiographic image-quality assessment, automated measurements, risk stratification, and prediction of clinical deterioration. In children with complex CHD, predictive models integrating physiological trends with anatomical diagnosis, surgical history, feeding and growth data, and previous clinical events could potentially identify patients at increased risk of interstage deterioration, unplanned admission, or other adverse events. AI may also assist clinicians in prioritizing the increasing volume of information generated by wearable and implantable devices by distinguishing potentially actionable events from artifacts or clinically insignificant alerts. Recent literature has highlighted the expanding role of AI across the continuum of congenital heart disease, including prenatal diagnosis and longitudinal management [5].

However, AI applications in pediatric cardiology require particularly careful validation. Algorithms trained predominantly on adult datasets may not generalize to children because cardiovascular physiology, normal reference ranges, congenital anatomy, disease prevalence, and device performance vary with age. Even within pediatric cardiology, small and heterogeneous patient populations may limit algorithm development. External validation in independent and representative pediatric cohorts is therefore essential before AI-supported systems are incorporated into routine care. Algorithmic bias, explainability, cybersecurity, data ownership, regulatory oversight, and clinical responsibility for AI-generated recommendations also require explicit consideration. AI should therefore be regarded as a clinical decision-support tool rather than an autonomous replacement for pediatric cardiology expertise.

Integration with EHRs will be equally important. At present, information generated by mobile applications, home-monitoring systems, wearable devices, and vendor-specific platforms may remain separated from the institutional medical record. Such fragmentation can increase clinician workload, create duplication, and increase the risk that important information is overlooked. Future systems should prioritize standardized and secure data exchange so that validated, clinically relevant information can be incorporated into the EHR and interpreted together with diagnoses, medications, imaging, laboratory results, and previous clinical encounters.

EHR integration may also support longitudinal visualization, structured documentation, automated risk stratification, and configurable alert pathways. However, successful integration should not simply maximize the volume of transmitted data. Excessive or poorly filtered information may contribute to alert fatigue and increase the burden on clinical teams. The priority should be identification of clinically actionable information, with clearly defined responsibility for review, appropriate response times, and escalation pathways. The future value of AI and predictive analytics will therefore depend not only on technical accuracy but also on their integration into clinically accountable and sustainable workflows.

### 5.4. Priorities for Future Research

The rapid evolution of digital technologies creates an additional challenge for evidence generation. Devices, software platforms, algorithms, and communication systems may change substantially during the time required to design, conduct, and publish a clinical trial. Consequently, some older studies remain clinically relevant because they evaluate fundamental models of care, such as remote echocardiographic interpretation or hub-and-spoke consultation, even though the original hardware may no longer reflect current practice. Conversely, newer technologies may enter clinical use before sufficient pediatric outcome data are available. Publication date alone should therefore not be used as a surrogate for evidence quality; methodological rigor, pediatric validation, clinical relevance of outcomes, and duration of follow-up remain more important.

Future research should move beyond demonstrating whether telemedicine is technically feasible and instead determine which interventions improve clinically meaningful outcomes, for which children, and under which organizational conditions. Prospective multicenter pediatric studies are required to improve statistical power and generalizability, particularly for uncommon congenital and acquired cardiovascular disorders. Studies should use clearly defined comparator pathways and standardized outcomes, including diagnostic accuracy, morbidity, adverse events, emergency visits, hospital admissions, transfers, quality of life, caregiver burden, healthcare utilization, and mortality where appropriate.

Pediatric-specific validation of wearable and home-monitoring technologies should include systematic assessment of signal quality, age-related performance, false-positive and false-negative findings, and the downstream consequences of device-generated alerts. Longer-term studies are needed to determine whether improvements in access, adherence, communication, or diagnostic yield ultimately translate into meaningful changes in clinical outcomes.

Economic evaluation and health-equity analysis should also be incorporated prospectively rather than considered secondary outcomes. Studies should document infrastructure costs, technical-support requirements, clinician workload, data-management burden, reimbursement mechanisms, and sustainability after pilot or research funding ends. Differences in access and outcomes according to socioeconomic status, geographical location, language, ethnicity, disability, digital literacy, and internet availability should be evaluated explicitly.

Future AI and predictive-analytics studies should use transparent methods, representative pediatric datasets, independent external validation, and clinically meaningful comparator pathways. Studies should assess not only model discrimination but also calibration, potential bias, false alerts, effects on clinician workload, and whether algorithm-supported decision-making improves outcomes over standard care.

Finally, telemedicine should be evaluated as a component of an integrated clinical pathway rather than as an isolated technology. The effectiveness of remote monitoring depends not only on the accuracy of the device but also on who reviews the generated information, how rapidly abnormalities trigger action, how families are educated, and how remote findings are integrated with conventional assessment. Recent pediatric telecardiology programs demonstrate the feasibility of connecting community pediatricians and tertiary pediatric cardiologists through structured digital systems [45]. The next phase of research should therefore focus on pragmatic implementation across diverse healthcare environments and determine how telemedicine, remote monitoring, AI-supported analysis, EHR integration, and face-to-face specialist care can operate as complementary components of a coordinated pediatric cardiovascular care pathway.

## 6. Conclusions

Telemedicine is becoming an increasingly important component of pediatric cardiovascular care, particularly for tele-echocardiography, monitoring of high-risk infants with CHD, remote blood pressure assessment, arrhythmia surveillance, and selected forms of longitudinal follow-up. Its principal value lies in extending specialist expertise beyond tertiary centers, facilitating communication with families and local professionals, and supporting earlier recognition of clinically relevant changes.

However, the maturity of the evidence differs considerably among applications. Tele-echocardiography and structured monitoring of selected high-risk populations have the strongest clinical experience, whereas many mobile-health and consumer wearable technologies remain supported predominantly by observational, single-center, or feasibility data. Their potential benefits must therefore be balanced against limited pediatric validation, false-positive and false-negative findings, data burden, privacy concerns, costs, and inequalities in digital access.

Accordingly, telemedicine should currently be viewed as a complement to appropriately organized pediatric cardiology care rather than a universal replacement for face-to-face assessment. Widespread implementation should be supported by secure interoperable infrastructure, trained personnel, clearly defined clinical responsibilities and escalation pathways, and equitable access for families.

Practical implementation of a pediatric cardiology telemedicine service requires considerably more than the availability of a videoconferencing platform. A sustainable program requires adequate hardware and network infrastructure, secure data storage and transmission, technical support, appropriately equipped clinical spaces, and sufficient clinical and administrative personnel to organize appointments, receive and review transmitted data, troubleshoot technical problems, and define pathways for escalation to face-to-face assessment. The technological solution may range from institutionally approved videoconferencing platforms and secure web links to dedicated telehealth applications that integrate physiological data, images, or ECG recordings. Consumer messaging or videoconferencing applications may be convenient and familiar to families, but their use must comply with local requirements for confidentiality, informed consent, data protection, documentation, and secure storage of clinical information. Whenever possible, communication and data transmission should therefore occur through institutionally approved platforms integrated with established clinical workflows and electronic health records.

Family engagement is equally important for successful implementation. Caregivers should receive clear instructions regarding how to access virtual consultations, operate home-monitoring devices, transmit recordings or measurements, recognize technical failures, and understand which symptoms require urgent conventional medical assessment rather than teleconsultation. Digital literacy, language, internet availability, access to suitable devices, and caregiver workload should be considered when selecting patients for remote care. Providing an initial demonstration, written or video instructions, technical contact support, and a clearly defined escalation pathway may improve adherence and reduce anxiety. Thus, telemedicine should be regarded as a structured clinical service supported by technology, personnel, training, governance, and family education rather than simply as remote communication between clinicians and patients.

The future of pediatric cardiology will depend on the thoughtful integration of digital health into routine clinical practice. When appropriately validated and embedded within structured care pathways, telemedicine has the potential to support families in the management of congenital and acquired cardiovascular diseases, improve communication with healthcare teams, and promote more timely, equitable, and personalized care for children and adolescents with heart disease.

## Figures and Tables

**Table 1:** Main applications of telemedicine in pediatric cardiology.

Clinical Area	Telemedicine Application	Target Population	Main Clinical Utility	Potential Benefits	Main Limitations
Fetal cardiology	Fetal tele-echocardiography	Pregnancies at risk of fetal congenital heart disease or fetal arrhythmias	Remote expert assessment of fetal cardiac anatomy and rhythm	Earlier diagnosis, optimized delivery planning, timely referral to tertiary centers	Dependence on image quality, operator expertise, and availability of adequate technology
Neonatal cardiology	Neonatal tele-echocardiography	Newborns with suspected congenital heart disease in peripheral hospitals or Level I-II NICUs	Remote interpretation of echocardiographic images by pediatric cardiologists	Improved triage, reduced unnecessary transfers, earlier identification of critical disease	Need for trained local personnel and standardized acquisition protocols
Congenital heart disease	High-risk infant home monitoring	Infants with complex CHD, especially single-ventricle physiology during the interstage period	Daily remote monitoring of oxygen saturation, weight, feeding, and clinical status	Earlier detection of deterioration, improved communication with families, potential reduction in interstage mortality	Data fragmentation, caregiver burden, and variable access to digital devices
Pediatric hypertension	Home blood pressure monitoring	Children and adolescents with suspected or confirmed hypertension	Repeated home measurements using validated devices and digital platforms	Reduced white-coat effect, improved diagnostic accuracy, safer follow-up and treatment titration	Need for validated pediatric devices, correct cuff size, and caregiver training
Arrhythmias	Smartphone-enabled ECG devices	Children with palpitations, syncope, suspected supraventricular tachycardia, or intermittent arrhythmias	On-demand ECG recording during symptoms	Improved symptom-rhythm correlation, earlier diagnosis, possible reduction in emergency visits	Limited pediatric validation, risk of false positives, and poor-quality tracings
Long-term follow-up	Virtual visits and remote consultations	Children and adolescents with stable cardiovascular disease requiring follow-up	Clinical assessment, review of symptoms, counseling, and triage	Reduced travel burden, improved continuity of care, better access to specialists	Not suitable for all clinical situations; physical examination and imaging may still be required

**Table 2:** Tele-echocardiography in congenital heart disease: models, advantages, and challenges.

Model	Description	Main Setting	Advantages	Challenges
Store-and-forward tele-echocardiography	Echocardiographic images are acquired locally and transmitted for later review by a pediatric cardiologist	Peripheral hospitals, outpatient clinics, neonatal units	Flexible scheduling, reduced need for immediate specialist availability, useful for screening	Delayed feedback; examination may need to be repeated if images are inadequate
Real-time tele-echocardiography	Images are acquired while the remote pediatric cardiologist provides live guidance to the local operator	NICUs, emergency settings, suspected critical CHD	Immediate interpretation, improved image acquisition, faster clinical decision-making	Requires stable internet connection and synchronized availability of local and remote teams
Hybrid tele-echocardiography	Combination of real-time guidance and asynchronous expert review	Level I-II NICUs and spoke centers without on-site pediatric cardiology	Balances flexibility with specialist support, improves triage and referral appropriateness	Requires structured workflow, trained personnel, and defined escalation pathways
Fetal tele-echocardiography	Remote evaluation of fetal cardiac anatomy or rhythm	Peripheral obstetric units, rural areas, high-risk pregnancies	Enhances prenatal detection, supports delivery planning, facilitates early referral	Requires high-quality fetal imaging and trained sonographers
Emergency tele-echocardiography	Rapid remote cardiac assessment in acutely ill neonates or children	Emergency departments and neonatal stabilization units	Supports urgent decisions regarding transfer, prostaglandin therapy, and intensive care	Time-sensitive and highly dependent on technical reliability and expert availability

**Table 3:** Digital tools for home monitoring and arrhythmia detection in children.

Digital Tool	Main Use	Parameters Collected	Pediatric Applications	Advantages	Limitations
Mobile health applications	Structured home monitoring and caregiver reporting	Symptoms, feeding, weight, oxygen saturation, blood pressure, medication adherence	Interstage monitoring in CHD, hypertension follow-up, chronic disease management	Easy data entry, automated alerts, longitudinal visualization of trends	Variable usability, need for caregiver engagement, privacy and interoperability concerns
Bluetooth pulse oximeters	Remote oxygen saturation monitoring	SpO2 and heart rate	Infants with single-ventricle physiology, cyanotic CHD, postoperative monitoring	Early detection of desaturation, useful in high-risk infants	Motion artifacts, incorrect probe placement, need for caregiver training
Digital scales	Growth and fluid status monitoring	Daily body weight	Infants with CHD, heart failure risk, interstage monitoring	Helps detect poor growth, dehydration, or fluid retention	Requires consistent measurement conditions and accurate data transmission
Home blood pressure monitors	Remote hypertension assessment	Systolic and diastolic blood pressure, heart rate	Children and adolescents with suspected or confirmed hypertension	Repeated measurements in real-life settings, reduced white-coat effect	Pediatric validation, correct cuff size, and interpretation by percentiles are required
Smartphone-enabled ECG devices	On-demand rhythm assessment	Single-lead ECG, heart rate, rhythm strip	Palpitations, syncope, supraventricular tachycardia, atrial arrhythmias	Captures intermittent arrhythmias during symptoms, facilitates remote review	Single-lead tracing may be insufficient; signal quality depends on correct use
Smartwatches	Rhythm screening and ECG recording in selected devices	Photoplethysmography, pulse irregularity alerts, single-lead ECG	Intermittent palpitations, follow-up of selected rhythm disorders	Widely available, enables patient-initiated recordings	Mostly designed for adults; false alerts and limited pediatric validation
Patch monitors	Prolonged ambulatory ECG monitoring	Continuous ECG over several days	Intermittent arrhythmias, unexplained symptoms, post-treatment monitoring	Longer recording duration than standard Holter, improved arrhythmia detection	Cost, skin irritation, data burden
Smart textiles	Continuous or intermittent ECG monitoring	ECG signals, heart rate, sometimes respiratory data	Long-term monitoring in children requiring repeated rhythm assessment	Potentially more comfortable than conventional Holter systems	Still under evaluation; limited availability and standardization
Implantable loop recorders	Long-term rhythm surveillance	Continuous/subcutaneous ECG with automatic and patient-triggered event storage	Recurrent unexplained syncope, infrequent palpitations, suspected paroxysmal arrhythmias	Very prolonged monitoring; captures events that may be missed by short-term or patient-activated devices; remote transmission possible	Requires minor invasive implantation; cost; false detections/data burden; specialist interpretation required

## Data Availability

No new data were created or analyzed in this study. Data sharing is not applicable to this article.
